# MicroRNAs as Important Regulators Mediate the Multiple Differentiation of Mesenchymal Stromal Cells

**DOI:** 10.3389/fcell.2021.619842

**Published:** 2021-06-07

**Authors:** Chao Yang, Maowen Luo, Yu Chen, Min You, Qiang Chen

**Affiliations:** ^1^Stem Cells and Regenerative Medicine Research Center, Sichuan Stem Cell Bank/Sichuan Neo-Life Stem Cell Biotech Inc., Chengdu, China; ^2^Center for Stem Cell Research and Application, Institute of Blood Transfusion, Chinese Academy of Medical Sciences, Peking Union Medical College, Chengdu, China

**Keywords:** microRNAs, menchymal stem/stromal cells, adipogenesis, chondrogenesis, osteogenesis

## Abstract

MicroRNAs (miRNAs) are endogenous short non-encoding RNAs which play a critical role on the output of the proteins, and influence multiple biological characteristics of the cells and physiological processes in the body. Mesenchymal stem/stromal cells (MSCs) are adult multipotent stem cells and characterized by self-renewal and multidifferentiation and have been widely used for disease treatment and regenerative medicine. Meanwhile, MSCs play a critical role in maintaining homeostasis in the body, and dysfunction of MSC differentiation leads to many diseases. The differentiation of MSCs is a complex physiological process and is the result of programmed expression of a series of genes. It has been extensively proven that the differentiation process or programmed gene expression is also regulated accurately by miRNAs. The differentiation of MSCs regulated by miRNAs is also a complex, interdependent, and dynamic process, and a full understanding of the role of miRNAs will provide clues on the appropriate upregulation or downregulation of corresponding miRNAs to mediate the differentiation efficiency. This review summarizes the roles and associated signaling pathways of miRNAs in adipogenesis, chondrogenesis, and osteogenesis of MSCs, which may provide new hints on MSCs or miRNAs as therapeutic strategies for regenerative medicine and biotherapy for related diseases.

## Introduction

In the late 1980s, mesenchymal stem/stromal cells (MSCs) were isolated from bone marrow and named, which was contrary to the theory that hematopoietic stem cells were the unique stem cell in bone marrow at that time (Friedenstein et al., 1987; Caplan, 1991). Later, apart from bone marrow, MSCs were isolated from a series of different tissue sources including adipose tissue, umbilical cord blood, umbilical cord, placenta, dental pulp, dental follicle, and so on (Erices et al., 2000; Gronthos et al., 2000; Zuk et al., 2001; In ‘t Anker et al., 2004; Kim et al., 2004; Morsczeck et al., 2005). Such cells have common biological characteristics such as strong proliferation capacity, self-renewal, and multidifferentiation. Since the success of obtaining induced pluripotent stem cells (iPSCs), iPSCs have become the new source to derive MSCs, and iPSC-derived MSCs display strong potential in angiogenesis, immunomodulation, and cell proliferation (Zhang et al., 2012; Liao et al., 2019). Nowadays, MSCs have been widely researched for disease treatment and regenerative medicine (Fitzsimmons et al., 2018). Previous studies reported that the therapeutic effect of MSCs is mainly through paracrine actions (Ding et al., 2018), including exosomes, microRNAs (miRNAs), mitochondria, and cytokines (Zhang et al., 2015; Spees et al., 2016; Jiang et al., 2019).

MiRNAs are endogenous single-strand non-encoding RNAs with the length of approximately 19–25 nucleotides, which are widely found in eukaryotic organisms (Bartel, 2009). Long primary miRNAs (pri-miRNAs) are transcribed from the genome and fold into hairpins. Through the cleavage by nuclease Drosha, the long pri-miRNA is converted to a miRNA precursor (pre-miRNA) of approximately 70 nucleotides. Pre-miRNAs are exported to the cytoplasm and act as the substrates of Dicer, a member of the RNase III family. The product of Dicer is a miRNA/miRNA^∗^ duplex about 20 base pairs. Generally, miRNA^∗^ is degraded, and the other strand of the duplex as the mature miRNA can be incorporated into an RNA-induced silencing complex (RISC). Depending on the sequence of miRNA, RISC can target the complementary sequence and bind to the messenger RNAs (mRNAs) (Krol et al., 2010). Finally, miRNAs influence the stability or translational efficiency of their target mRNAs, which results in downregulated gene expression or protein output (Baek et al., 2008; Mohr and Mott, 2015). At present, more than 1,000 miRNAs have been discovered to regulate a series of biological processes, including proliferation, differentiation, development, apoptosis, and so on (Krol et al., 2010; Dexheimer and Cochella, 2020).

In the last few years, miRNAs have been widely reported to play a critical role in the differentiation of MSCs, including both positive and negative regulation. This positive or negative regulation may be essential for the application of different therapeutic strategies in tissue regenerative medicine or for a better understanding of the molecular mechanisms of human diseases related to MSC differentiation. This review discusses recent research progress on the roles and mechanisms of miRNAs in adipogenesis, chondrogenesis, and osteogenesis of MSCs, which may provide new theoretical or clinical hints on regenerative medicine and the treatment for related diseases.

## MiRNAs Regulate the Adipogenesis of MSCs

**FIGURE 1:**
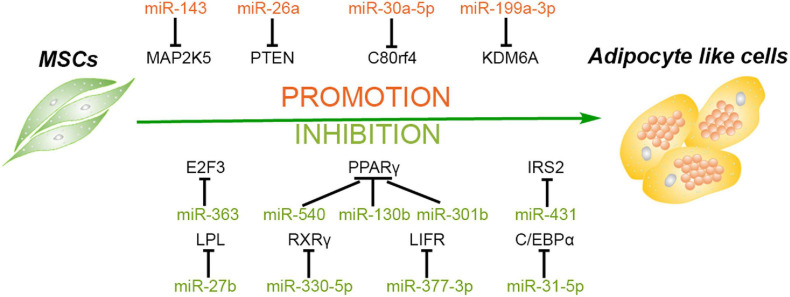
Roles of different miRNAs in regulating adipogenesis of mesenchymal stem cells. Some miRNAs promote the adipogenesis of mesenchymal stem/stromal cells (MSCs) *via* silencing the expression of MAP2K5, PTEN, C80rf4, and KDM6A, while some miRNAs inhibit this process through targeting the critical factors and their activators of adipogenesis, including PPARγ, C/EBPα, RXRγ, E2F3, and so forth.

In the era of stem cell biology, adipogenic differentiation is considered as one of the criteria for defining MSCs (Dominici et al., 2006). Meanwhile, adipogenesis is an important physiological process to produce adipocytes for energy homeostasis and also related to many diseases (Liu et al., 2017). For instance, the adipocyte hyperplasia and abnormal metabolism of adipose tissue may lead to obesity, which is considered to be the major risk of many associated metabolic complications and chronic diseases (Schuster, 2010; Longo et al., 2019). Adipose-derived stem cells (ADSCs) play a critical role in the generation and metabolism of adipose tissues; thus, investigation and regulation of the adipogenic differentiation of ADSCs is necessary to understand and treat associated diseases (Silva and Baptista, 2019). Additionally, it has been reported that miRNAs are involved in the adipogenic differentiation of MSCs and even play a pivotal role in obesity-associated diseases (Hamam et al., 2015; Zaiou et al., 2018). MiRNAs and their targets in the adipogenesis of MSCs are summarized in Figure 1.

### MiRNAs Promote the Adipogenesis of MSCs

The adipogenic differentiation process of MSCs contains three main stages. First, MSCs are committed into progenitors and subsequently differentiate into preadipocytes. Second, the preadipocytes begin to reach confluence, and the growth is arrested. Finally, the cells manifest adipocyte morphology and express specific genes of adipocyte, including peroxisome proliferator-activated receptor gamma (PPARγ), CCAAT/enhancer binding protein alpha (C/EBPα), and so on (Gregoire et al., 1998). A previous study demonstrated that miR-143 is downregulated in the initial 2 days and upregulated in the following 5 days of adipogenesis of ADSCs, suggesting that the levels of miR-143 are not constant in adipogenic differentiation. A further investigation revealed that miR-143 inhibits adipogenesis during the clone expansion stage by blocking DNA synthesis and mitotic division *via* directly targeting MAP2K5 mRNA, while downregulation of MAP2K5 promotes adipogenic differentiation *via* attenuating the phosphorylation of PPARγ in the terminal differentiation stage (Hu et al., 1996; Chen et al., 2014b). Another study demonstrated that miR-26a and miR-26b are upregulated during the adipogenic differentiation of human ADSCs, and miR-26a increases the number of adipocytes with the marker of thermogenic adipocytes uncoupling protein 1 (UCP1) and changes the mitochondrial phenotype, suggesting that miR-26a promotes human ADSCs to differentiate toward brown adipocytes (Karbiener et al., 2014). In addition, miR-26a also mediated this adipogenic differentiation of MSCs derived from other sources. Trohatou et al. (2017) demonstrated that miR-26a plays a positive role in the adipogenic differentiation of amniotic fluid MSCs (AFMSCs) *via* targeting phosphatase and tensin homolog (PTEN) and, in turn, promotes the phosphorylation of Akt and the expression of its downstream mammalian target of rapamycin (mTOR). This study also confirmed that miR-26a can also modulate cell cycle through inhibiting the expression of Cyclin E1 and Cyclin-dependent kinase 6 (CDK6), demonstrating that miR-26a may induce cell cycle arrest at the second differentiation stage (Trohatou et al., 2017).

Wnt signaling pathway and histone methylation have been reported to play crucial roles in the regulation of differentiation of MSCs, including inhibition of adipogenesis (Cawthorn et al., 2012; Yuan et al., 2016). As an epigenetic regulator of histone H3 lysine 27 (H3K27), lysine demethylase 6A (KDM6A) has been demonstrated to repress the adipogenesis of bone marrow MSCs (BMSCs) *via* activating the Wnt pathway. MiR-199a-3p continues to be upregulated during the adipogenic differentiation process of BMSCs, and miR-199a-3p mimics enhance the adipogenesis of BMSCs by binding to the 3′ untranslated region (UTR) of KDM6A, in turn suppressing the activation of Wnt signaling (Shuai et al., 2019). Besides short non-coding RNAs, the long non-coding RNAs (lncRNAs) can also competitively bind to the miRNAs to regulate cellular differentiation. Li et al. (2019a) demonstrated that lncRNA H19 and miR-30a-5p negatively regulate each other through direct binding. Meanwhile, miR-30a-5p directly binds to the 3′ UTR of C8orf4, leading to enhanced expression of PPARγ and CEBPα and lipid accumulation in human ADSCs (Jang et al., 2016. Hence, a competitive relationship is formed between lncRNA H19 and C8orf4, and lncRNA H19 attenuates the adipogenic differentiation of human ADSCs. MiRNAs enhancing adipogenic differentiation of MSCs and their target genes are listed in Table 1.

**TABLE 1:** MicroRNAs involved in adipogenesis of MSCs.

miRNAs	Target genes/pathways	MSC types	Promotion/inhibition of adipogenesis	References
miR-363	E2F3	Rat ADSCs	Inhibition	Chen et al., 2014a
miR-143	MAP2K5, MAPK pathway	Rat ADSCs	Promotion	Chen et al., 2014b
miR-540	PPARγ	Human subchondral MSCs	Inhibition	Chen et al., 2015
miR-301b/miR-130b/miR-330-5p	PPARγ	Human UCMSCs, BMSCs, ADSCs/Rat MSCs	Inhibition	Liu et al., 2017; Huang et al., 2018
miR-26a	PTEN	Human amniotic fluid MSCs	Promotion	Trohatou et al., 2017
miR-27b	LPL	Human ADSCs	Inhibition	Hu et al., 2018
miR-377-3p	LIFR	Human BMSCs	Inhibition	Li et al., 2018
miR-31-5p	C/EBPα	Human ADSCs	Inhibition	Liu Y. et al., 2018
miR-431	IRS2	Human BMSCs	Inhibition	Wang et al., 2018
miR-30a-5p	C8orf4	Human ADSCs	Promotion	Li et al., 2019a
miR-199a-3p	KDM6A, Wnt pathway	Mouse BMSCs	Promotion	Shuai et al., 2019

### MiRNAs Inhibit the Adipogenesis of MSCs

Many miRNAs inhibiting the adipogenic differentiation of MSCs were decreased during this process. As mentioned above, the process of adipogenesis contained three main stages. MiR-363 is one of the significantly decreased miRNAs in adipogenic differentiation progress, and miR-363 blocks the transition from clonal expansion to terminal differentiation *via* targeting E2F3, a key factor controlling the cell cycle and adipogenesis, resulting in the suppression of the adipogenesis in ADSCs (Chen et al., 2014a). As one of the classical adipocyte-specific genes and critical transcription factor of adipogenesis, PPARγ ensures the maintenance of terminal adipogenic differentiation and is highly expressed in adipose tissues (Lehrke and Lazar, 2005). In addition, miR-540 is also downregulated persistently during adipogenic differentiation, and PPARγ is the main binding target of miR-540 (Chen et al., 2015). Another study compared the adipogenic capacity among ADSCs, BMSCs, and UCMSCs and confirmed that PPARγ expression displays a significant positive correlation with their adipogenic capacity. Additionally, this study also demonstrated that both miR130b and miR301b suppress adipogenesis by directly targeting PPARγ as well, and their expression negatively correlates with that of PPARγ in MSCs from three sources (Liu et al., 2017). Previous studies reported that miR-27a and miR-27b have a negative effect on PPARγ and C/EBPα, and confirmed that PPARγ is one of their direct targets, which is consistent with the pivotal role of PPARγ in the inhibition of adipogenesis and lipid metabolism (Gu et al., 2016; Hsu et al., 2017; Seenprachawong et al., 2018). Later, Hu et al. (2018) demonstrated that lipoprotein lipase (LPL) is also the direct target of miR-27b in the adipogenesis of ADSCs, suggesting that miR-27b can be a candidate target for regulating triglyceride *in vivo*.

In addition to PPARγ itself, its activators, such as retinoid X receptor γ (RXRγ), are also the target of miRNAs to suppress the adipogenesis process. RXRγ is a member of the nuclear receptor superfamily and expressed in brown adipocytes (Svensson et al., 2014). One study reported that the expression of RXRγ and PPARγ is upregulated while the expression of miR-330-5p is downregulated in the adipogenic differentiation of rat MSCs induced by H_2_O_2_. MiR-330-5p directly targets the 3′ UTR of RXRγ to inhibit the expression of PPARγ and C/EBPα, lipogenic genes such as adipocyte fatty acid-binding protein (aP2), and glucose transporter 4 (GLUT4) (Huang et al., 2018).

As another important transcription factor participating in adipogenic differentiation, C/EBPα cross-regulates with PPARγ to govern the expression of the entire process of adipogenesis, inducing activation of additional transcription factors and production of the adipocyte phenotype (Farmer, 2005; Tang and Lane, 2012). MiR-31-5p can directly bind to the 3′ UTR of C/EBPα and hinder the adipogenesis of human ADSCs. Moreover, C/EBPα can activate the expression of lncRNA terminal differentiation-induced ncRNA (TINCR) *via* binding to its promoter region, which inhibits the expression of miR-31-5p. Therefore, lncRNA TINCR, miR-31-5p, and C/EBPα may form a feedback loop to mediate the adipogenesis process of human ADSCs (Liu Y. et al., 2018). In addition to PPARγ and C/EBPα, other target genes involved in glucose, adipose, and bone metabolism also play important roles in adipogenic differentiation. For instance, leukemia inhibitory factor (LIF) and its receptor (LIFR) have been reported to play important roles in osteoblast, osteoclast, chondrocyte, and adipocyte differentiation (Sims and Johnson, 2012). MiR-377-3p is significantly decreased during the adipogenesis of human BMSCs, and overexpression of miR-377-3p attenuates the expression of adipogenic markers and suppresses the adipogenesis process through directly targeting LIFR (Li et al., 2018). Furthermore, insulin receptor substance 2 (IRS2), as one of the most ubiquitously expressed members of the IRS family, plays a vital role in the metabolic actions of glucose and adipocyte differentiation (Schinner et al., 2005). The expression of miR-431 is also decreased during the adipogenic process of human BMSCs. Overexpression of miR-431 inhibits the expression of PPARγ and C/EBPα and is accompanied by the repression of the adipogenesis process. Later, it was confirmed that miR-431 binds to the 3′ UTR of IRS2 to achieve the inhibition effect (Wang et al., 2018). MiRNAs suppressing the adipogenic differentiation of MSCs and their target genes are summarized in Table 1.

## MiRNAs Mediate the Osteogenesis of MSCs

**FIGURE 2:**
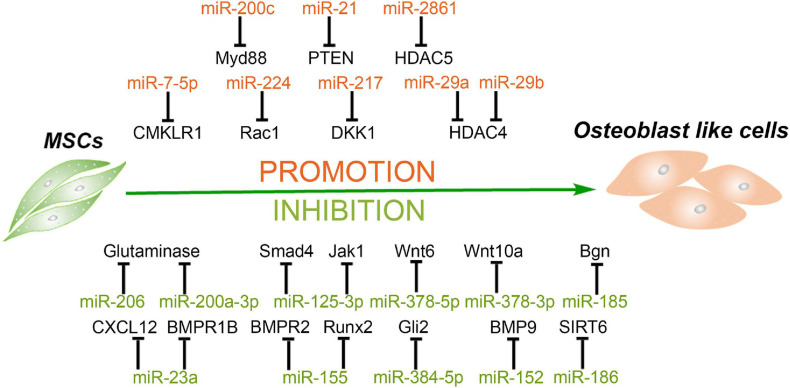
MiRNAs mediate the osteogenic differentiation of mesenchymal stem cells. MiRNAs promote the osteogenesis of MSCs *via* silencing the expression of HDAC4/5, CMKLR1, DKK1, Myd88, PTEN, etc. and in turn inhibit histone deacetylation, activation of Wnt/β-catenin, and Akt signaling. Meanwhile, some miRNAs inhibit the osteogenesis of MSCs through targeting vital regulators, synergistic enhancer, or essential nutrients in this process, including BMPs, BMPRs, Gli2, SIRT6, glutamine, and so forth.

One previous study demonstrated that osteoblasts are differentiated from BMSCs *in vivo* and are one of the pivotal cell types participating in bone formation (Wang J. et al., 2019). Dysfunction of osteogenesis of BMSCs can lead to many diseases, such as osteoporosis and other diseases associated with bone loss. Meanwhile, MSCs are an important source of seed cells for bone tissue engineering and have been used in clinics (Ferrarotti et al., 2018). Therefore, understanding the mechanisms of osteogenesis and the regulation of the osteogenic differentiation of MSCs is very important. MiRNAs as important regulators of differentiation are also involved in the osteogenesis of MSCs. MiRNAs and their targets in the osteogenesis of MSCs are summarized in Figure 2.

### MiRNAs Enhance the Osteogenesis of MSCs

Histone acetylation is an epigenetic process, with histone acetyltransferases and histone deacetylases (HDACs) acting antagonistically on chromatin modification and transcription. HDAC4 is a class IIa HDAC implicated in many biological processes, including bone formation (Sild and Booij, 2019). MiR-29a promotes osteogenic differentiation of the SMSCs *via* directly targeting HDAC4 and Wnt3a signaling, and overexpressed miR-29a in osteoblasts leads to a high bone mass in the subchondral region of transgenic mice, suggesting miR-29a is a positive regulator for osteogenesis and bone formation (Lian et al., 2017). Lately, another study reported that a cell-penetrating peptide named R9-LK15 can form a nanocomplex system to delivery miR-29b to BMSCs efficiently. R9-LK15/miR-29b nanocomplexes enhance the osteogenesis of BMSCs also *via* targeting HDAC4 (Liu Q. et al., 2019). In addition, HDAC5, as a negative regulator of runt-related transcription factor 2 (Runx2), is a target of miR-2861. Knockdown of miR-2861 inhibits bone formation and decreases bone mass *in vivo*, suggesting that miR-2861 also plays a positive role in osteogenesis (Li et al., 2009).

Wnt pathway activation has been reported to increase bone formation and decrease resorption. Chemokine-like receptor 1 (CMKLR1) and dickkopf-related protein 1 (DKK1) as the inhibitors or responsive genes of the Wnt/β-catenin pathway regulate the osteogenesis of MSCs (Evenepoel et al., 2015; Muruganandan et al., 2017). MiR-7-5p is gradually elevated during the osteogenic differentiation process of human BMSCs, and miR-7-5p mimics enhance this process *via* binding to the 3′ UTR of CMKLR1 (Chen et al., 2018). Dai et al. (2019) observed that miR-217 expression is markedly downregulated in steroid-associated osteonecrosis patients. MiR-217 promotes the nuclear translocation of β-catenin and, in turn, the proliferation and osteogenic differentiation of MSCs by targeting DKK1. In addition, Rac family small GTPase 1 (Rac1), as a member of the GTPase family, interacts with DKK1/Wnt/β-catenin signaling and serves as an important target in the osteogenic differentiation process (Wu et al., 2008; Onishi et al., 2013). Cai et al. (2019) revealed that miR-224 is upregulated in osteoblastic differentiation of human MSCs, and miR-224 mimic improves alkaline phosphatase (ALP) activity and matrix mineralization *via* targeting Rac1 and subsequently regulates the JAK/STAT and Wnt/β-catenin signaling to enhance osteogenesis.

Besides the Wnt pathway, Akt signaling also regulates bone growth and formation. Overexpression of miR-200c cannot only enhance the osteogenic differentiation of human BMSCs but also promote the proliferation of osteoblasts by activating the Akt/β-catenin signaling pathway *via* targeting Myd88, which plays a positive role in inflammation and fibrosis but a negative one in osteoblastic differentiation (Xia et al., 2019). In addition, miR-21 overexpression promotes the migration and osteogenic differentiation of dog BMSCs *in vitro* by enhancing HIF-1α activity *via* the PTEN/PI3K/Akt pathway. Furthermore, miR-21-modified BMSCs can exhibit greater potential for new bone formation compared with wild-type BMSCs (Yang et al., 2019). MiRNAs facilitating the osteogenic differentiation of MSCs and their target genes are summarized in Table 2.

**TABLE 2:** MicroRNAs involved in osteogenesis of MSCs.

miRNAs	Target genes/pathways	MSCs	Promotion/inhibition of osteogenesis	References
miR-29a/miR-29b	HDAC4	Human subchondral MSCs/rat BMSCs	Promotion	Lian et al., 2017; Liu Y. et al., 2019
miR-2861	HDAC5	Mouse BMSCs (ST2 cells)	Promotion	Li et al., 2009
miR-7-5p	CMKLR1	Human BMSCs	Promotion	Chen et al., 2018
miR-224	Rac1	Human AMMSCs	Promotion	Cai et al., 2019
miR-206/miR-200a-3p	Glutaminase	Human BMSCs	Inhibition	Chen et al., 2019; Lv et al., 2019
miR-185	Biglycan, BMP/Smad pathway	Mouse BMSCs	Inhibition	Cui et al., 2019
miR-217	DKK1	Human BMSCs	Promotion	Dai et al., 2019
miR-125a-3p	Smad4 and Jak1	Human ADSCs	Inhibition	Gu et al., 2019
miR-152	BMP9, PI3K/AKT, Wnt/β-catenin pathways	Rat BMSCs	Inhibition	Li et al., 2019b
miR-384-5p	Gli2	Rat BMSCs	Inhibition	Li et al., 2019c
miR-30b	Runx2	Rat BMSCs	Inhibition	Liu Q. et al., 2019
miR-200c	Myd88, AKT/β-catenin pathway	Human BMSCs	Promotion	Xia et al., 2019
miR-21	PTEN, PI3K/Akt/HIF-1α pathway	Human UCBMSCs	Promotion	Yang et al., 2019
miR-23a	BMPR1B, CXCL12	Human PDLSCs, BMSCs	Inhibition	Zhang et al., 2019; Zhuang et al., 2019
miR-186	SIRT6	Human BMSCs	Inhibition	Xiao et al., 2020
miR-378-3p/miR-378-5p	Wnt10a/Wnt6a	Human BMSCs/mouse BMSCs	Inhibition	Feng et al., 2020

### MiRNAs Attenuate the Osteogenesis of MSCs

Bone morphogenetic proteins (BMPs), members of the transforming growth factor beta (TGF-β) superfamily, have been confirmed as vital regulators in bone formation and can influence the osteogenic differentiation of MSCs. BMP2 is an important regulator in the early stage of osteogenesis. One previous study reported that biglycan (Bgn), an extracellular matrix proteoglycan in the small leucine-rich proteoglycans (SLRP) family, significantly enhances the BMP-2-induced Smad1/5/9 phosphorylation and osteogenic differentiation in mouse myogenic C2C12 cells (Jongwattanapisan et al., 2018). Cui et al. (2019) demonstrated that both primary osteoblasts and MSCs derived from miR-185-knockout (KO) mice have better osteogenesis capacity than cells from wild-type mice and confirmed that Bgn is the direct target of miR-185. Moreover, compared with wild-type mice, more bone formation has been detected in miR-185-KO mice (Cui et al., 2019). In addition, overexpressed miR-155 in MSCs decreases ALP activity and Alizarin red S staining and downregulates the expression of osteogenic markers during the osteogenesis process induced by BMP9. Runx2 and BMP receptor 2 (BMPR2) are two direct target genes of miR-155 (Liu H. et al., 2018).

BMP receptors are essential for BMPs to activate the phosphorylation of Smad1/5/9 and in turn to promote osteogenesis. In addition to BMPR2 mentioned above, BMPR type 1B (BMPR1B) has also been reported to be vital for osteogenesis. Zhang et al. (2019) found that miR-23a increases in periodontal MSCs (PDLSCs) of chronic periodontitis patients and inhibits the osteogenesis of PDLSCs. Further investigation indicated that miR-23a targets BMPR1B to inhibit the phosphorylation of Smad1/5/9 and the BMP pathway (Zhang et al., 2019). However, BMPR1B is not the only target of miR-23a to inhibit osteogenesis. Another study demonstrated that miR-23a inhibits nanotube Ti-induced osteogenic differentiation of human BMSCs *via* targeting the C-X-C motif ligand 12 (CXCL12). CXCL12, also named stromal cell derived factor-1 (SDF-1) and as the ligand of C-X-C chemokine receptor type 4 (CXCR4), participates in osteogenic differentiation of MSCs (Li et al., 2017; Zhuang et al., 2019).

Besides BMP2, BMP9 is another potent member in BMPs to promote ALP activity and osteogenic differentiation of MSCs (Liu H. et al., 2018). One previous study has reported that BMP9 can enhance osteogenesis *via* canonical Wnt/β-catenin signaling pathway (Tang et al., 2009). MiR-152 is downregulated during *Astragalus* polysaccharide-induced osteogenesis of rat BMSCs, and BMP9 is the direct target of miR-152. Overexpression of miR-152 also inhibits the proliferation and osteogenic differentiation of BMSCs *via* the BMP9/PI3K/AKT and Wnt/β-catenin pathways (Li et al., 2019b). Furthermore, a growth hormone has been reported to synergize with BMP9 to facilitate osteogenic differentiation through the JAK/STAT/IGF1 pathway, and Jak1 participates in the process of osteoblasts (Huang E. et al., 2012). A significant decrease in miR-125a-3p level during osteogenic differentiation of human ADSCs was observed. Luciferase reporter assay and western blot confirmed that both Smad4 and Jak1 are direct targets of miR-125a-3p (Gu et al., 2019).

The Indian hedgehog could exhibit a synergistic effect with BMP2 to promote osteogenic potential of human MSCs (Reichert et al., 2013), and Gli2 is a transcriptional activator that plays a crucial role in Indian hedgehog signaling pathway to promote osteoblast development and cartilage vascularization (Joeng and Long, 2009). MiR-384-5p is obviously upregulated in BMSCs from aged rats as compared with that from young rats. Overexpression of miR-384-5p in BMSCs from young rats suppresses osteogenesis and accelerates senescence, whereas downregulation of miR-384-5p in BMSCs from aged rats has the opposite effects, suggesting that miR-384-5p plays a vital role in cell senescence and bone loss caused by aging. Further investigation confirmed that miR-384-5p achieves the above inhibition effect by directly binding to the 3′ UTR of Gli2 mRNA (Li et al., 2019c). In addition, Sirtuin6 (SIRT6), as an H3K9 deacetylase, has been demonstrated to enhance the osteogenesis of human BMSCs and bone formation *via* BMP signaling (Zhang et al., 2017). MiR-186 upregulation significantly suppresses the osteogenic differentiation of human BMSCs *via* targeting the 3′ UTR of SIRT6 (Xiao et al., 2020).

Of note, activation of the Wnt/β-catenin pathways is vital for osteogenesis and bone formation. Wnt family members can be the targets to attenuate osteogenesis. Feng et al. (2020) demonstrated that abnormal bone tissues and impaired bone quality can be observed in miR-378 transgenic mice, and a delayed healing effect is also observed during bone fracture. The osteogenesis of the BMSCs from transgenic mice is also suppressed. Further investigation confirmed that miR-378-5p and miR-378-3p target Wnt6 and Wnt10a, respectively, to inactivate Wnt/β-catenin signaling, resulting in the suppression of osteogenesis and impairment of bone formation (Feng et al., 2020). Moreover, miR-378a has been reported to contribute to impairment of tenogenic differentiation and tendon injuries *in vitro* and *in vivo*, respectively. TGF-β2 was identified as the target of miR-378a both in mouse and human (Liu Y. et al., 2019).

Glutamine is the most abundant non-essential amino acid in the body. Meanwhile, it can be used to synthesize glutamate and subsequently converted into α-ketoglutarate. α-Ketoglutarate is an essential nutrient involved in the osteogenesis of BMSCs and bone formation (Karner et al., 2015). Therefore, glutamine metabolism could be a target of miRNAs to inhibit the osteogenic differentiation of MSCs. MiR-206 is gradually downregulated during the process of human BMSC osteogenic differentiation. Overexpression of miR-206 attenuates ALP activity, osteocalcin secretion, and inhibits glutamine metabolism *via* directly binding to the 3′ UTR of glutaminase mRNA. Moreover, miR-200a-3p is highly expressed in the serum of osteoporosis patients and downregulated during the osteogenic differentiation of MSCs. The ALP activity, calcification nodule, osteogenic markers and even cell viability are suppressed by overexpressed microRNA-200a-3p. Finally, dual-luciferase reporter gene assay showed that glutaminase is also the direct target of miR-200a-3p (Lv et al., 2019). MiRNAs suppressing the osteogenic differentiation of MSCs and their target genes are summarized in Table 2.

## MiRNAs Modulate the Balance of Adipogenesis and Osteogenesis

**FIGURE 3:**
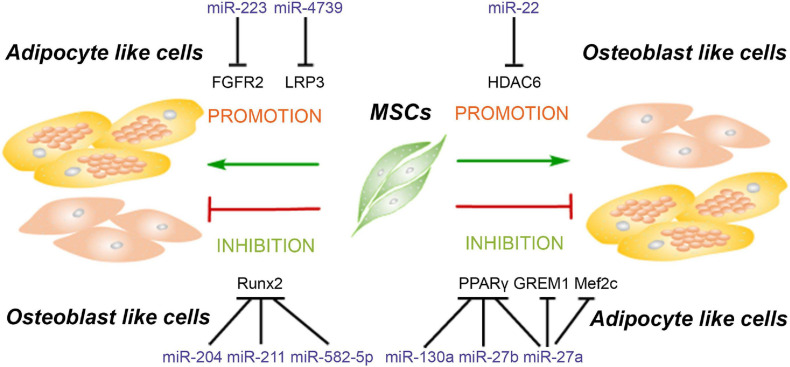
MiRNAs modulate the balance of adipogenesis and osteogenesis of mesenchymal stem cells. MiRNAs promote the adipogenesis and attenuate osteogenesis though targeting Runx2, FGFR2, and LRP3. Meanwhile, some miRNAs enhance the osteogenesis and suppress adipogenesis of MSCs *via* inhibiting the expression of PPARγ, GREM1, Mef2c, and HDAC6.

One previous study reports that an imbalanced adipogenic and osteogenic differentiation has been considered as a reason for age-related impairment of bone formation (Elsafadi et al., 2017). Indeed, adipogenesis and osteogenesis relating to fat and bone formation in the human body are inversely correlated (Greco et al., 2015), and many previous studies have shown that the interactional lncRNAs and miRNAs have an opposite effect on the adipogenesis and osteogenesis of MSCs (Guan et al., 2015; Huang et al., 2015, 2016; Gu et al., 2016; Elsafadi et al., 2017). MiRNAs and their targets involved in regulating the balance of adipogenesis and osteogenesis of MSCs are summarized in Figure 3.

### MiRNAs Promote Adipogenesis and Inhibit Osteogenesis of MSCs

Runx2 as one of the classical osteogenic markers and key transcription factor regulating osteogenesis has become a common target for several miRNAs to inhibit osteogenesis, for instance, miR-155 as mentioned above. Moreover, Runx2 silencing not only inhibits the osteogenesis but also promotes the adipogenesis of MSCs. Huang et al. (2010) reported that miR-204 and its homolog miR-211 are upregulated in mesenchymal progenitor cell lines and rat BMSCs during adipogenic differentiation. Upregulation or downregulation of miR-204 accompanies the corresponding downregulation or upregulation of its target Runx2, relating to promotion of osteogenesis and inhibition of adipogenesis or promotion of adipogenesis and inhibition of osteogenesis, respectively (Huang et al., 2010). Another study showed that miR-582-5p is associated with the pathology of subchondral bone sclerosis and involved in osteoarthritis. Further investigation revealed that miR-582-5p promotes osteogenesis and attenuates the adipogenesis of mesenchymal progenitor C3H10T1/2 cells also *via* targeting Runx2 (Wang P. et al., 2019).

Besides Runx2, fibroblast growth factor receptor (FGFR) and low-density lipoprotein receptor-related protein 3 (LRP3) are also reported to be the targets of miRNAs to participate in the regulation of adipogenic and osteogenic differentiation. MiR-223 is upregulated in mouse BMSCs, C3H10T1/2 mesenchymal cell line, and ST2 stromal cell line after adipogenic-induced differentiation, but reduced in preosteoblast MC3T3-E1 cell line after osteogenic differentiation. Meanwhile, miR-223 mimics inhibit the proliferation and induce cells to differentiate into adipocytes *via* targeting FGFR2. Downregulation of FGFR2 inhibits the phosphorylation of ERK1/2 and upregulates the expression of C/EBPα. It is interesting that C/EBPs also induce the expression of miR-223 to form a C/EBPs/miR-233/FGFR2 feedback loop and regulates the balance of adipogenesis and osteogenesis (Guan et al., 2015). The LRP family members as endocytic receptors mediate the uptake of lipoproteins, the roles of which are well-appreciated in many physiological processes, including lipid metabolism, cardiac disease, tumorigenesis, and so on (Kang, 2020). As one member of the LRP family, LRP3 expression levels in the highly osteogenic human BMSC clone are higher than those in the weakly osteogenic human BMSC clone. Meanwhile, miR-4739 is the most underrepresented miRNA in these two cell clones. Overexpression of miR-4739 promotes adipogenic and suppresses osteogenic differentiation of human BMSCs *via* targeting LRP3 (Elsafadi et al., 2017). MiRNAs suppressing the osteogenic and enhancing adipogenic differentiation of MSCs and their target genes are summarized in Table 3.

**TABLE 3:** MicroRNAs involved in modulating the balance of adipogenesis and osteogenesis of MSCs.

miRNAs	Target genes/pathways	MSC types	Pro-adipogenesis/anti-osteogenesis	Anti-adipogenesis/pro-osteogenesis	References
miR-204/miR-211/miR-582-5p	Runx2	BMSCs	√		Huang et al., 2010; Wang P. et al., 2019
miR-22	HDAC6	Human ADSCs		√	Huang S. et al., 2012
miR-223	FGFR2	Mouse BMSCs	√		Guan et al., 2015
miR-27a	PPARγ/GREM1/Mef2c	Rat BMSCs		√	Gu et al., 2016; You et al., 2016
miR-4739	LRP3	Human BMSCs	√		Elsafadi et al., 2017
miR-130a/miR-27b	PPARγ	Human BMSCs		√	Seenprachawong et al., 2018

### MiRNAs Enhance Osteogenesis and Suppress Adipogenesis of MSCs

As mentioned above, PPARγ is a critical transcription factor for adipogenesis and has been reported to be the target of miRNAs which suppress adipogenic differentiation of MSCs. Recent studies reveal that knockdown of PPARγ by miRNAs not only inhibits adipogenesis but also promotes osteogenesis. MiR-27a is downregulated and negatively correlated with PPARγ and gremlin 1 (GREM1) expression in steroid-induced osteonecrosis of femoral head patients. A further study reveals that both PPARγ and GREM1 are the direct target genes of miR-27a, and upregulation of miR-27a impairs adipogenesis and enhanced osteogenesis in steroid-induced rat BMSCs, suggesting that miR-27a is a potential target for the treatment of steroid-induced osteonecrosis of the femoral head (Gu et al., 2016). Additionally, miR-130a and miR-27b enhance the osteogenesis of human BMSCs also *via* targeting PPARγ (Seenprachawong et al., 2018). Although this study did not investigate the effect of these two miRNAs on the adipogenesis of human BMSCs, it is reasonable to postulate that adipogenesis will be dampened since PPARγ is the major transcription factor for adipogenesis. Moreover, miR-27a is significantly reduced in osteoporotic patients, and miR-27a increased osteogenesis and decreased adipogenesis *via* targeting myocyte enhancer factor 2C (Mef2c), a transcription factor related to chondrocyte hypertrophy and bone development (Arnold et al., 2007; You et al., 2016). These results suggested that upregulation of miR-27a may be a potential therapeutic strategy for the treatment of bone degeneration diseases.

Previous studies have reported that HDAC4 and HDAC5 are targeted by miR-29a/b and miR-2861, resulting in the enhancement of osteogenesis in MSCs (Lian et al., 2017; Liu Q. et al., 2019). In addition to HDAC4, other HADC family members including HDAC6 have also been demonstrated to interact with Runx2 and inhibit the process of osteogenesis (Li et al., 2009). Huang S. et al. (2012) reported that miR-22 is decreased during adipogenesis but increased during osteogenesis of human ADSCs, and the results from multiple sources have proved that HDAC6 is the direct target gene of miR-22, suggesting that miR-22 is a critical regulator to mediate the balance between adipogenesis and osteogenesis of hADMSCs by silencing HDAC6. MiRNAs enhancing the osteogenic and attenuating adipogenic differentiation of MSCs and their target genes are summarized in Table 3.

Although it is not explicitly stated in these articles, besides the miRNAs mentioned in Figure 3, we can also speculate that miRNAs mediating the adipogenesis in Figure 1 may also play inverse roles in osteogenesis, while miRNAs mediating the osteogenesis in Figure 2 also played inverse roles in adipogenesis. For instance, Chen et al. (2018) demonstrated that miR-7-5p targets CMKLR1 to enhance osteogenesis without mentioning the effect on adipogenesis, but another study has confirmed that CMKLR1 knockdown in MSCs not only enhances osteogenesis but also inhibits adipogenesis of MSCs (Muruganandan et al., 2017).

## MiRNAs Regulate the Chondrogenesis of MSCs

**FIGURE 4:**
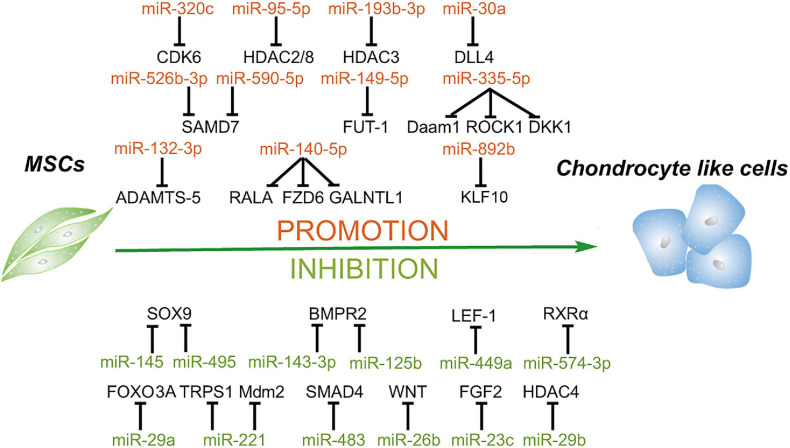
Roles of miRNAs in chondrogenic differentiation of mesenchymal stem cells. MiRNAs enhance the chondrogenesis of MSCs *via* downregulating the expression of the negative regulators of chondrogenesis, activators of Notch and NF-κB signaling, enhancer of chondrocyte hypertrophy, including RALA, FZD6, GALNTL1, DLL4, HDAC4, SMAD7, KLF10, etc. In addition, some miRNAs suppress this process through targeting key factors of chondrogenesis, including SOX9, BMPR2, FOXO3A, HDAC4, SMAD4, and so on.

The damage of articular cartilage cannot be repaired spontaneously (Karlsen et al., 2014). So far, many clinical strategies have been attempted to repair the cartilage lesions; however, no treatment has so far obtained the desired outcomes. At present, cell-based therapy is widely used for treating diseases, including autologous chondrocyte implantation for cartilage repair (Roberts et al., 2003; Minas et al., 2010). However, the chondrocytes do not express type II collagen (COL2) and Aggrecan (ACAN) of hyaline cartilage but secretes type I collagen (COL1) and versican of fibrocartilage when cultured as monolayer, which would limit the clinical outcomes (Knutsen et al., 2007; Malicev et al., 2009); instead, the chondrogenic differentiation of MSCs with scaffold may be a potential treatment strategy to overcome this limitation (Patrascu et al., 2013). Likewise, microRNAs are thought to be involved in cartilage development, homeostasis, and disease (Mirzamohammadi et al., 2014; Swingler et al., 2019) and actually also play an essential role in chondrogenic differentiation of MSCs, both positive and negative (Chen et al., 2019; Lee et al., 2019; Shen et al., 2019). MiRNAs and their targets in the chondrogenesis of MSCs are summarized in Figure 4.

### MiRNAs Facilitate the Chondrogenesis of MSCs

Karlsen et al. (2014) showed that miR-140-5p and miR-140-3p are most highly expressed in chondrogenic differentiating MSCs and uncultured articular chondrocytes. Moreover, this study confirms that a small GTPase named RAS like proto-oncogene A (RALA) is a direct target of miR-140-5p, and inhibition of miR-140 results in the downregulation of the sex-determining region Y-box transcription factor 9 (SOX9) and ACAN (Karlsen et al., 2014). Thereafter, another study also found that miR-140-5p is an important positive regulator of chondrocyte gene expression and represses multiple negative regulators of chondrogenesis, including frizzled class receptor 6 (FZD6), polypeptide *N*-acetylgalactosaminyltransferase 1 (GALNTL1), and also RALA. Additionally, this study identifies that miR-140-5p has a positive regulation effect on the Wnt signaling pathway, which plays an essential role in chondrogenesis and skeletal development (Barter et al., 2015). Lin et al. (2014) reported that miR-335-5p and its host gene Mesoderm-specific transcript (Mest) are upregulated during the chondrogenic differentiation of mouse MSCs. Moreover, miR-335-5p targets disheveled-associated activator of morphogenesis 1 (Daam1) and Rho-associated coiled-coil containing protein kinase 1 (ROCK1), the negative regulators of SOX9, to downregulate miR-29a and miR-29b. More interestingly, both miR-29a and miR-29b are involved in the downregulation of Mest. Meanwhile, miR-335-5p also targets DKK1, a Wnt signaling pathway inhibitor, to increase the expression of Mest through the β-catenin/TCF signaling pathway. Therefore, miR-335-5p and its host gene Mest form two positive feedback loops to promote chondrogenic differentiation of mouse MSCs (Lin et al., 2014).

Previous studies demonstrated that activation of Notch signaling attenuates chondrogenic differentiation of MSCs, and delta-like 4 (DLL4) is a membrane-bound ligand of Notch signaling family, suggesting that DLL4 may play a negative role in chondrogenic differentiation (Jiang et al., 2013; Tian et al., 2015). Upregulation of miR-30a remarkably promotes the chondrogenic differentiation of rat MSCs and increases the expression of COL2 and ACAN through directly targeting DLL4 (Tian et al., 2016). HDACs, as central regulators for the stability of chromatin structure and epigenetic regulation, are also involved in the initiation and progression of osteoarthritis (Furumatsu and Ozaki, 2010; Bradley et al., 2015; Carpio and Westendorf, 2016). For instance, HDAC1, 2, 3, and 8 repress the development of cartilage through inhibiting the expression of cartilage-specific genes, and HDAC4 inhibits the expression of Runx2 to regulate hypertrophy, chondrogenesis, and skeletogenesis (Vega et al., 2004; Hong et al., 2009; Mao et al., 2018; Meng et al., 2018; Zhao et al., 2019), indicating that the majority of HDACs may play a negative role in chondrogenesis. A recent study reported that overexpression of miR-95-5p is essential for chondrogenesis through suppressing the expression of HDAC2/8, which plays an essential role in the expression of cartilage-specific genes, including collagen type II alpha 1 chain (COL2A1), ACAN, and SOX9 (Mao et al., 2018). Moreover, another study showed that miR-193b-3p promotes histone H3 acetylation in promoters of COL2A1, ACAN, cartilage oligomeric matrix protein (COMP), and SOX9 by targeting HDAC3 and, thus, facilitates the chondrogenesis and hypertrophy of human BMSCs. Additionally, this study also confirmed that miR-193b overexpression can strongly enhance cartilage formation *in vivo* (Meng et al., 2018).

Interleukin-1β (IL-1β), as one of the most common proinflammatory cytokines, suppresses the synthesis of proteoglycan and collagen and increases significantly in chondrocytes and synoviocytes of osteoarthritis patients, suggesting that IL-1β plays a crucial role in osteoarthritis development (Wang et al., 1997; Kapoor et al., 2011). Previous studies showed that miR-320c is involved in and regulates inflammation (Meng et al., 2016; Pierdomenico et al., 2016). Overexpression of miR-320c can attenuate the expression of inflammatory factors induced by IL-1β through binding to the 3′ UTR of CDK6 *via* the NF-κB signaling pathway (Sun et al., 2018). As proinflammatory cytokines play a vital role in osteoarthritis development, inhibition of inflammation and promotion of chondrogenesis have become the potential strategies to relieve osteoarthritis. Melatonin as an efficient antioxidant has been confirmed to have anti-inflammatory effect through inhibiting the activation of the NF-κB pathway (Cuesta et al., 2010; Moniruzzaman et al., 2018). Meanwhile, melatonin facilitates the chondrogenesis of human BMSCs (Gao et al., 2014). A recent study further demonstrated that melatonin promotes chondrogenic process *via* the upregulation of miR-526b-3p and miR-590-5p, which enhances SMAD1 phosphorylation through targeting SMAD7. Thus, this study speculates that melatonin treatment or miR-526b-3p and miR-590-5p transduction may be an effective therapy for cartilage degeneration (Wu et al., 2018). Besides inflammatory cytokines, a series of aggrecanases also facilitate the development of osteoarthritis, such as a disintegrin and metalloproteinase with thrombospondin motifs (ADAMTS) family (Yang et al., 2017). ADAMTS-5 has been reported to have the highest activity in this family to accelerate the development of osteoarthritis (Verma and Dalal, 2011; Apte, 2016). Zhou et al. (2018) revealed that overexpression of miR-132-3p can enhance the chondrogenic differentiation of rat MSCs by targeting ADAMTS-5, indicating that miR-132-3p may be a new therapeutic for patients with osteoarthritis.

From the above, miRNAs have the potential to be the therapeutic agents for osteoarthritis treatment, and researchers speculate that some agents such as nanoparticles may improve the delivery efficiency of miRNAs. Nanoparticles with a concentration gradient of hyaluronic acid (HA) and chitosan (CS) can be prepared from HA/CS with varying concentrations of chondroitin sulfate solution *via* ionic interaction. MiR-149-5p was loaded with these HA/CS nanoparticles and incubated with human MSCs. Transfection of miR-149-5p through this delivery system successfully enhances the chondrogenic differentiation by targeting fucosyltransferase-1 (FUT-1) (Celik et al., 2019). In addition, as one of the major pathological factors in osteoarthritis, chondrocyte hypertrophy is the terminal stage of chondrocyte differentiation and responsible for the formation of endochondral bone (Zhao et al., 2019). Kruppel-like factor 10 (KLF10) has been reported to be a critical and positive regulator for bone mineralization in osteoblasts and may be involved in chondrocyte hypertrophy. MiR-892b can inhibit hypertrophy through mediating the cross-talk between TGF-b/Smad and Indian hedgehog signaling by targeting KLF10 (Lee et al., 2019). MiRNAs facilitating the chrondrogenic differentiation of MSCs and their target genes are summarized in Table 4.

**TABLE 4:** MicroRNAs involved in chondrogenesis of MSCs.

miRNAs	Target genes/pathways	MSCs	Promotion/inhibition of chondrogenesis	References
miR-145/miR-495	SOX9	Mouse MSCs/human BMSCs	Inhibition	Yang et al., 2011; Lee et al., 2014
miR-449a	LEF-1	Human BMSCs	Inhibition	Paik et al., 2012
miR-574-3p	RXRα	Human BMSCs	Inhibition	Guerit et al., 2013
miR-29a	FOXO3A	Human BMSCs	Inhibition	Guerit et al., 2014
miR-140-5p	RALA/FZD6/GALNTL1, Wnt pathway	Human BMSCs	Promotion	Karlsen et al., 2014; Barter et al., 2015
miR-335-5p	Daam1/ROCK1/DKK1, Wnt/β-catenin/TCF pathway	Mouse MSCs	Promotion	Lin et al., 2014
miR-221	TRPS1/Mdm2	Human UCMSCs and BMSCs	Inhibition	Lolli et al., 2016
miR-30a	DLL4, Notch signaling	Rat BMSCs	Promotion	Tian et al., 2016
miR-483	SMAD4	Human BMSCs	Inhibition	Anderson and McAlinden, 2017
miR-95-5p	HDAC2/8	Human BMSCs	Promotion	Mao et al., 2018
miR-193b-3p	HDAC3	Human BMSCs	Promotion	Meng et al., 2018
miR-320c	CDK6	Human BMSCs	Promotion	Sun et al., 2018
miR-143-3p/miR-125b	BMPR2	Rat BMSCs	Inhibition	Tian et al., 2018; Xiao et al., 2019
miR-526b-3p/miR-590-5p	SAMD7	Human BMSCs	Promotion	Wu et al., 2018
miR-132-3p	ADAMTS-5	Rat BMSCs	Promotion	Zhou et al., 2018
miR-149-5p	FUT-1	Human MSCs	Promotion	Celik et al., 2019
miR-26b	Wnt	Rat BMSCs	Inhibition	Huang et al., 2019
miR-892b	KLF10, TGF-β/SMAD and Ihh pathway	Human BMSCs	Promotion	Lee et al., 2019
miR-23c	FGF2	Rat BMSCs	Inhibition	Shen et al., 2019
miR-29b	HDAC4	Mouse MSCs	Inhibition	Zhao et al., 2019

### MiRNAs Suppress the Chondrogenesis of MSCs

It is well known that SOX9 is a master positive regulator of chondrogenesis and also affects the chondrogenic differentiation of MSCs; thus, it can be the target of miRNAs to inhibit chondrogenesis. MiR-145 is downregulated during TGF-β3-induced chondrogenesis of mouse MSCs, and overexpression of miR-145 suppresses chondrogenic differentiation by directly targeting SOX9 (Yang et al., 2011). MiR-495 also represses the chondrogenic differentiation of human BMSCs *via* binding the 3′ UTR of SOX9 (Lee et al., 2014). Besides targeting SOX9, miRNAs also affect some other important signal pathways to suppress chondrogenesis. For instance, Wnt signaling has been reported to play a critical role during the process of chondrogenesis (Chun et al., 2008). A recent study found that miR-26b can inhibit the activation of the Wnt/β-catenin signaling pathway *via* directly targeting Wnt and ultimately suppress the chondrogenic differentiation of rat BMSCs (Huang et al., 2019). Additionally, Wnt3a exerts a positive effect on chondrogenesis *via* β-catenin/lymphoid enhancer-binding factor-1 (LEF-1) to upregulate the expression of SOX9 (Fischer et al., 2002). Paik et al. (2012) reported that miR-449a directly binds to the 3′ UTR of LEF-1, ultimately leading to the reduction of SOX9 expression and suppression of chondrogenesis.

As mentioned before, the miR-29 family consists of critical positive regulators for osteogenesis (Lian et al., 2017), while this family plays a negative role in chondrogenesis. Overexpression of miR-29a suppresses the differentiation of human BMSCs toward chondrocytes by targeting fork head box O3A (FOXO3A). FOXO3A is a positive regulator of SOX9, and SOX9 plays a negative role in miR-29a expression (Guerit et al., 2014). Also as mentioned before, miR-29b suppresses the expression of HDAC4, and HDAC4 has been demonstrated to have an inhibition effect on chondrocyte hypertrophy by repressing the activity of Runx2 (Vega et al., 2004), resulting to enhance osteogenesis. In addition, miR-221 has been reported to play an antichondrogenic role in both human BMSCs and Wharton’s jelly-derived MSCs, and a further study indicates that miR-221 blocks cartilage synthesis *via* targeting mouse double-minute 2 homolog (Mdm2) and transcriptional repressor GATA binding 1 (TRPS1) (Lolli et al., 2016). Downregulation of TRPS1 induces cartilage hypertrophy and extracellular matrix (ECM) degradation *via* Runx2 signaling, while inhibition of the expression of Mdm2 suppresses the cartilage ECM synthesis through prevention of the degradation of Slug, which is a protein upregulated in osteoarthritic chondrocytes (Lisignoli et al., 2014; Piva et al., 2015; Lolli et al., 2016). Thus, suppressing miR-29 and miR-221 expression may be necessary for articular cartilage repair, and the balance of SOX9 and Runx2 determines the differentiation fate of chondrocytes.

Bone morphogenetic proteins are also involved in chondrogenic differentiation, endochondral bone formation, and embryogenesis (Tsumaki and Yoshikawa, 2005; Cheng et al., 2016). BMP receptors are transmembrane serine/threonine kinases (Liu et al., 1995), and BMPR2 plays a vital role in a series of biological processes, including proliferation, apoptosis, and osteogenetic and chondrogenic differentiation (Zhou et al., 2016; Kovermann et al., 2019). MiR-143-3p is decreased during the early phase of chondrogenesis of rat BMSCs, and miR-143-3p can repress the deposition of proteoglycans and the expression of chondrogenic markers *via* directly targeting BMPR2 (Tian et al., 2018). In addition, miR-125b is upregulated in osteoarthritis cartilages. A further study confirms that miR-125b can inhibit chondrogenic differentiation and promotes apoptosis of human BMSCs also through targeting BMPR2 (Xiao et al., 2019). In addition, SMAD4 is also involved in both TGF-β signaling and BMP signaling *via* SMAD2/3 and SMAD1/5/8 and required for MSC chondrogenesis *in vitro* (Hellingman et al., 2011). MiR-483 is upregulated in human and mouse osteoarthritis tissues, and miR-483 also represses the expression of SOX9 and chondrogenesis process in part by targeting SMAD4 (Anderson and McAlinden, 2017). In addition, as one of the most wildly used growth factors in the differentiation of MSCs, fibroblast growth factor 2 (FGF2) enhances the proliferation and suppresses the cell senescence in the chondrogenesis of MSCs (Correa et al., 2015). Furthermore, FGF2 also regulates the immediate response of articular cartilage to mechanical injury (Vincent et al., 2002). However, Shen et al. (2019) reported that miR-23c is downregulated during chondrogenic induction, and overexpression of miR-23c can suppress the differentiation toward chondrocytes through targeting FGF2. MiRNAs inhibiting the chondrogenic differentiation of MSCs and their target genes are summarized in Table 4.

### Dual Characters of MiRNAs and Homeostasis in the Chondrogenesis of MSCs

Although MSCs from many tissue sources have been proposed as potential candidates for cartilage repair, the MSCs from a specific tissue, such as the synovium, may have a unique advantage for cartilage regeneration (Jones and Pei, 2012). The combination of synovium-derived MSCs and miRNAs is speculated to possibly obtain better clinical outcomes. However, a previous study demonstrates that miR-218 overexpression significantly enhances chondrogenic differentiation of synovium MSCs by upregulating the expression of chondrogenic markers (SOX9, COL2A1, ACAN, and COMP) in early stage, but inhibits maturation *via* suppressing the expression of maturation markers (CMP, COL10A1, MMP-13, and VEGF) through targeting 15-hydroxyprostaglandin dehydrogenase (HPGD) (Chen et al., 2019). This result indicates that some miRNAs may not play consistently positive roles in the chondrogenic differentiation of MSCs and cartilage repair. Similarly, another study reported that the expression of miR-574-3p is increased during the early phase of BMSC chondrogenic differentiation with Retinoid X receptor alpha (RXRα), an inhibitor of chondrogenesis, as its direct target (Weston et al., 2002; Guerit et al., 2013). However, while overexpression of miR-574-3p inhibits chondrogenic differentiation *in vitro*, either upregulation or downregulation of miR-574-3p expression represses the formation of cartilage and endochondral bone in a heterotopic ossification model (Guerit et al., 2013). This study suggested that there may be a dynamic balance between the expression levels of miR-574-3p and its target, and a threshold level of RXRα is required for adult MSC chondrogenesis. This implies that disruption of the homeostasis of the expression of certain miRNAs may have a negative effect on chondrogenesis and cartilage formation.

In summary, miRNAs play important roles in inhibiting targets during the chondrogenesis of MSCs. Only after we fully understand the role of miRNAs may an appropriate upregulation or downregulation of corresponding miRNAs be used to improve the chondrogenesis of MSCs and guide cartilage tissue engineering.

## The Application Strategy of MiRNAs Alone or Combined With MSC-Based Therapy

MiR-122 inhibitors were applied in a clinical trial to treat HCV infection (Janssen et al., 2013), indicating that miRNA mimics or inhibitors may be used also for the treatment of metabolic disorders and used as regenerative medicine directly. For instance, miR-26a promotes human ADSCs to differentiate toward brown adipocytes during the differentiation process, while activation of energy-consuming brown adipocytes is currently considered as a potential therapeutic approach to combat obesity (Volke and Krause, 2021). Thus, upregulation of the level of miR-26a *via* miRNA mimics or transcription activators may be a candidate therapy for obesity. Additionally, it has been reported that miR-200a-3p is highly expressed in the serum of osteoporosis patients and inhibits osteogenesis; thus, miR-200a-3p inhibitors may be used for the treatment of osteoporosis in the future. For cartilage repair, miR-193b overexpression can strongly enhance cartilage formation *in vivo*, while miR-29 and miR-221 inhibit articular cartilage repair; therefore, miR-193b mimics and miR-29 and miR-221 inhibitors may play positive roles for the treatment of cartilage degeneration.

To date, MSCs alone or combined with scaffold have been used in the treatment of multiple diseases and tissue engineering due to their regeneration and immunomodulation capacity. MiRNA mimics or inhibitors could also be included in MSC-based therapy to improve clinical outcomes, for instance increasing or decreasing miRNAs to promote the directional differentiation of MSCs before transplantation. A previous study demonstrated that osteo-differentiated BMSCs showed higher potential concerning *in vivo* bone regeneration compared with undifferentiated cells (Ye et al., 2012), suggesting that osteogenically differentiated MSCs regulated by miRNA mimics or inhibitors can be used to improve the outcomes of bone tissue engineering.

In addition, osteoarthritis is one of the representative cartilage diseases, and it is a chronic degenerative joint disease characterized by progressive cartilage deterioration, leading to the loss of joint function. However, current clinical therapeutic methods including surgical and pharmacologic treatment cannot regenerate the cartilage. MSCs combined with scaffold or factors have been used for the treatment of OA in preclinical and clinical studies. Previous studies demonstrated that transplantation with MSCs with an appropriate period of prechondrogenic differentiation acquires better outcomes (Wu et al., 2020), indicating that chondrogenic differentiation of MSCs *via* transfecting with prochondronic miRNAs or downregulating antichondronic miRNAs before transplantation may produce better MSCs for the treatment of OA.

Although miRNAs can regulate the adipogenic, osteogenic, and chondrogenic differentiation of MSCs, they can also regulate a series of other biological processes, including proliferation, development, apoptosis, and even tumorigenesis. As mentioned above, miR-199-3p plays a positive role in promoting the adipogenesis of BMSCs *via* targeting KDM6A and inactivating Wnt signaling. However, MSCs have been demonstrated to induce an increase of miR-199 and miR-214 to promote metastasis and maintain cancer stem cell phenotype of breast cancer cells (Cuiffo et al., 2014). In addition, miR-21 promotes the migration and osteogenic differentiation of BMSCs, and miR-21-overexpressed MSCs exhibit greater potential for new bone formation *in vivo*. MiR-21 is also an overexpressed oncogenic gene in triple-negative breast cancer and contributes to the downregulation of tumor suppressors, and the delivery of anti-miR-21 using RNA nanoparticles targeting cancer stem cells expressing CD133 resulted in the inhibition of tumor growth (Yin et al., 2019). Therefore, the application of miRNA-transfected MSCs or miRNA mimics or inhibitors should be considered carefully to avoid the promotion of tumorigenesis, drug resistance, tumor progression, and metastasis (Papaccio et al., 2017).

In addition, one miRNA could bind to serval targets and influence differentiation of MSCs and even multiple physiological processes, while the same gene also can be targeted by multiple miRNAs. This feature indicates that upregulation or downregulation of miRNAs does not affect only a single physiological process but also may be associated with some pathological alternations. If miRNA-transfected MSCs or miRNA mimics or inhibitors themselves are to be used or considered as therapeutic strategies for the treatment of related diseases, several problems should be addressed. First, although lots of miRNAs have been found to change during the multiple differentiation of MSCs, the focus must be on the miRNAs with significant up- or downregulation and meaningful targets. Second, whether regulating a single miRNA can achieve the aim of mediating differentiation should be noted because differentiation is a complex process, and the key differentiation genes might be targeted by more than one miRNA. Third, miRNA has been reported to reverse the differentiation of MSCs. MiR-378 has been demonstrated to promote BMP2-induced osteoblast differentiation in C2C12 cells (Hupkes et al., 2014). However, a recent study showed that impaired bone quality and a delayed healing effect were observed in the miR-378 transgenic mice, and the osteogenic differentiation of BMSCs from this transgenic mouse was also inhibited (Feng et al., 2020). Thus, the function of miRNAs should be identified carefully, and *in vivo* data are more important than *in vitro*. Fourth, to determine the safety of miRNA-transfected MSCs or miRNA mimics or inhibitors, as many physiological changes as possible should be evaluated and researched. In general, although the preclinical findings are just preliminary with a long way from benches to clinic, transplantation with directional differentiated MSCs or direct application of miRNA mimics or inhibitors may have a bright future after the mechanisms of action are fully understood, and the safety and stability are ensured through further studies.

## Conclusion

It is well known that MSCs play a critical role in maintaining homeostasis in the body. Dysfunction of MSC differentiation leads to many diseases, including obesity, osteoporosis, osteoarthritis, and so forth. Meanwhile, miRNAs play critical roles in the differentiation of MSCs.

We have provided a brief overview of the roles of miRNAs in MSC differentiation. First, miRNAs inhibit directional differentiation though targeting the critical factors and their activators of this process. For instance, PPARγ, C/EBPα, or their activators have been silenced by miRNAs to inhibit adipogenesis, and the receptors, the synergistic enhancers of BMPs, or themselves have been targeted to suppress osteogenesis. Second, the multiple differentiation of MSCs regulated by miRNAs is not entirely an isolated event. In other words, a miRNA exhibiting a regulatory effect on osteogenesis may show an opposite effect on adipogenesis or chondrogenesis. For example, miR-27a targets PPARγ to promote osteogenesis and inhibit adipogenesis; miR-29 targeting HDAC4 enhances osteogenesis but attenuates chondrogenesis. Third, plenty of essential signaling pathways and even cell cycle are regulated by miRNAs. For example, Wnt/β-catenin and Akt signaling are inhibited by miRNAs to attenuate osteogenic differentiation of MSCs, Notch and NF-κB signaling are hindered to suppress the chondrogenic differentiation of MSCs, and the cell cycle is blocked by miRNA *via* targeting CDK6 to promote adipogenic differentiation of MSCs. Fourth, one miRNA can bind to several targets and influence multiple differentiation of MSCs and even multiple physiological processes, while the same gene also can be targeted by multiple miRNAs. For instance, PPARγ is the target of miR-130a, miR-27a, and miR-27a, while GREM1 and Mef2C are both the target genes of miR-27a. Fifth, histone methylation and acetylation are implicated in MSC differentiation regulated by miRNAs. Silencing KDM6A by miRNA promotes adipogenic differentiation, targeting HADC4/5/6 promotes osteogenic differentiation, and binding of miRNAs to HDAC2/3/8 enhances chondrogenic differentiation. Finally, some miRNAs may not play consistent roles in the differentiation of MSCs. For example, miR-218 overexpression significantly enhances chondrogenic differentiation of MSCs in the early phase, but then inhibits the maturation process of chondrogenesis. Therefore, the multiple differentiation of MSCs regulated by miRNAs is a complex, interdependent, and dynamic process. MiRNA mimics/inhibitors and directional differentiated MSCs may become effective therapeutic agents for related diseases and powerful materials for tissue engineering. Thus, a full understanding of the roles of miRNAs is necessary and will provide hints on the appropriate upregulation or downregulation of corresponding miRNAs to improve clinical outcomes.

## Author Contributions

CY and QC conceived the manuscript. CY performed the data curation, wrote the original draft, and prepared the figures. ML, YC, and MY revised the manuscript for content. All authors approved the final version of the manuscript.

## Conflict of Interest

CY, ML, YC, MY, and QC are employed by company Sichuan Stem Cell Bank/Sichuan Neo-Life Stem Cell Biotech Inc. The authors declare that the research was conducted in the absence of any commercial or financial relationships that could be construed as a potential conflict of interest.
